# Extracellular Release of HMGB1 as an Early Potential Biomarker for the Therapeutic Response in a Xenograft Model of Boron Neutron Capture Therapy

**DOI:** 10.3390/biology11030420

**Published:** 2022-03-10

**Authors:** Shoji Imamichi, Lichao Chen, Tasuku Ito, Ying Tong, Takae Onodera, Yuka Sasaki, Satoshi Nakamura, PierLuigi Mauri, Yu Sanada, Hiroshi Igaki, Yasufumi Murakami, Minoru Suzuki, Jun Itami, Shinichiro Masunaga, Mitsuko Masutani

**Affiliations:** 1 Department of Molecular and Genomic Biomedicine, School of Biomedical Sciences, Nagasaki University Graduate, Nagasaki 852-8523, Japan; simamich@ncc.go.jp (S.I.); chen202107@outlook.com (L.C.); y-tong@nagasaki-u.ac.jp (Y.T.); takae-o@nagasaki-u.ac.jp (T.O.); jj20210059@ms.nagasaki-u.ac.jp (Y.S.); 2 Lab of Collaborative Research, Division of Cellular Signaling, National Cancer Center Research Institute, Tokyo 104-0045, Japan; j8313605@ed.tus.ac.jp; 3 Central Radioisotope Division, National Cancer Center Research Institute, Tokyo 104-0045, Japan; 4 Division of BNCT, EPOC, National Cancer Center, Tokyo 104-0045, Japan; satonaka@ncc.go.jp (S.N.); hirigaki@ncc.go.jp (H.I.); jitami@ncc.go.jp (J.I.); 5 Department of Biological Science and Technology, Faculty of Industrial Science and Technology, Tokyo University of Science, Tokyo 125-8585, Japan; yasufumi@rs.noda.tus.ac.jp; 6 Department of Radiation Oncology, National Cancer Center Hospital, Tokyo 104-0045, Japan; 7 Clinical Proteomics Laboratory, Institute of Biomedical Technologies, National Research Council, 93-20054 Milan, Italy; pierluigi.mauri@itb.cnr.it; 8 Institute for Integrated Radiation and Nuclear Science, Kyoto University, Kumatori 590-0494, Japan; sanada.yu.6n@kyoto-u.ac.jp (Y.S.); suzuki.minoru.3x@kyoto-u.ac.jp (M.S.); masunaga.shinichiro.6m@kyoto-u.jp (S.M.)

**Keywords:** ^10^B-boronophenylalanine (BPA), boron neutron capture therapy (BNCT), boron neutron capture reaction (BNCR), high mobility group box 1 (HMGB1)

## Abstract

**Simple Summary:**

Boron neutron capture therapy (BNCT) is a non-invasive therapeutic technique for treating malignant tumors, however, there is a lack of methods to evaluate its therapeutic efficacy. Herein, we investigated whether high mobility group box 1 (HMGB1) is a potential biomarker of BNCT response in tumor cells and mice in combination with ^10^B-p-boronophenylalanine in the Kyoto University Nuclear Reactor. We observed an increased extracellular HMGB1 release in cancer cells 24 h post-BNCT irradiation, compared to that observed with an equivalent dose of gamma-ray irradiation. High levels of plasma HMGB1 were observed on day 3 post-BNCT irradiation in a xenograft mouse model. These levels were stably detected even when the size of the tumor decreased, suggesting that HMGB1 is a potential biomarker for the therapeutic efficacy of BNCT.

**Abstract:**

Boron neutron capture therapy (BNCT) is a non-invasive therapeutic technique for treating malignant tumors, however, methods to evaluate its therapeutic efficacy and adverse reactions are lacking. High mobility group box 1 (HMGB1) is an inflammatory molecule released during cell death. Therefore, we aimed to investigate HMGB1 as a biomarker for BNCT response, by examining the early responses of tumor cells to ^10^B-boronophenylalanine (BPA)-based BNCT in the Kyoto University Nuclear Reactor. Extracellular HMGB1 release was significantly increased in human squamous carcinoma SAS and melanoma A375 cells 24 h after neutron irradiation but not after γ-irradiation. At 3 days post-BPA-based BNCT irradiation in a SAS xenograft mouse model, plasma HMGB1 levels were higher than those in the non-irradiation control, and HMGB1 was detected in both nuclei and cytoplasm in tumor cells. Additionally, increased plasma HMGB1 levels post-BNCT irradiation were detected even when tumors decreased in size. Collectively, these results indicate that the extracellular HMGB1 release occurs at an early stage and is persistent when tumors are reduced in size; therefore, it is a potential biomarker for evaluating the therapeutic response during BNCT.

## 1. Introduction

Boron neutron capture therapy (BNCT), a non-invasive radiotherapeutic technique, was developed for the selective treatment of cancer using compounds that contain a stable boron isotope ^10^B. BNCT is based on nuclear reactions between thermal neutrons and boron-10 atoms (boron neutron capture reaction, BNCR) that are preferentially distributed in cancer cells [1,2,3]. These nuclear reactions result in the release of alpha particles and recoiling lithium nuclei with short lengths that induce DNA damage. Several biomarkers for early and late responses to BNCT have been identified. Early BNCT response is evaluated using biomarkers for double strand breaks (DSB) and DNA damage, such as micronuclei formation [4,5], γH2AX foci [6,7], and 53BP1 foci formation and poly(ADP-ribose) [8]. Additionally, comprehensive proteomic analysis has been carried out in human oral cancer SAS cells using ^10^B-p-boronophenylalanine (BPA) as the boron delivery agent to identify intracellular early response markers for BNCT. This analysis showed that dynamic changes in the fragments of endoplasmic reticulum-localized lymphoid-restricted protein (LRMP) are a potential biomarker for the cellular responses to BNCT [9]. However, there is still a need for more effective biomarkers for the evaluation of cellular responses to BNCT. We have previously reported an early increase in the high mobility group box 1 (HMGB1) protein level in lymphosarcoma-bearing rats in response to BPA-based BNCT [8]. HMGB1 is a conserved non-histone DNA-binding protein that is ubiquitously expressed in cells [10] and belongs to the damage-associated molecular pattern (DAMP) group that promotes inflammation [11,12,13]. It is normally localized in the nucleus and regulates DNA stability by loosely binding to DNA [14]. HMGB1 is a proinflammatory ligand that binds to the receptor for advanced glycation end-products (RAGE) and Toll-like receptor (TLR) [15,16,17]. It is also involved in the DNA damage response and cell death [8,18,19]. Apoptotic, autophagic, and necrotic cell deaths result in the extracellular release of HMGB1 [12,20,21], which is associated with inflammation, sepsis, and autoimmune diseases [15]. Additionally, an elevated HMGB1 expression level has been reported in cancer cells. Therefore, this study aimed to investigate HMGB1 extracellular release from tumor cells during BPA-based BNCT. We demonstrated that the extracellular release of HMGB1 increased after neutron beam irradiation, thereby highlighting its potential as an early biomarker for tumor response to BNCT.

## 2. Materials and Methods

### 2.1. Cell Culture

Human melanoma A375 (ATCC, Manassas, VA, USA) and human squamous cell carcinoma SAS cell lines [22] were cultured in DMEM containing 10% fetal bovine serum (FBS), 1% penicillin and streptomycin (PS), and DMEM/Ham’s F12 medium containing 10% FBS and 1% PS, respectively. Both cell lines were cultured in a humidified CO_2_ incubator with 5% CO_2_ at 37 °C. The ^10^B-enriched (>98%) BPA was purchased from Katchem spol. s.r.o. (Prague, Czech Republic). The BPA fructose complex was prepared and added to the cell culture, as previously described [9,23,24].

### 2.2. Irradiation

**Figure 1:**
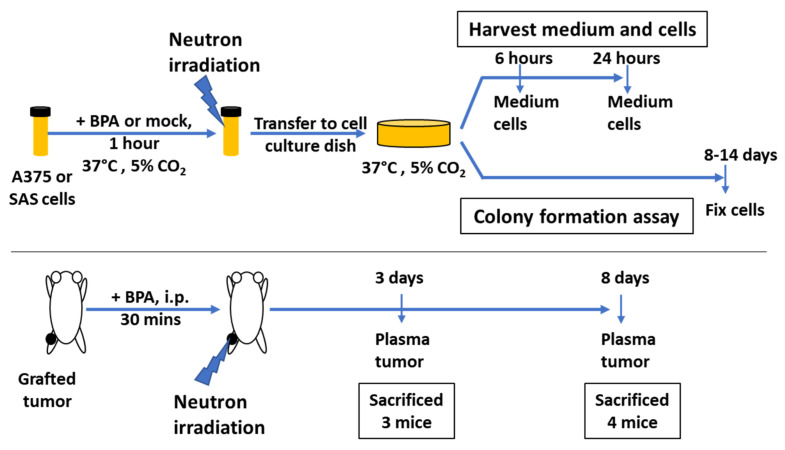
Schematic diagram of cell and mouse irradiation experiments. Cell suspensions were subjected to neutron beam irradiation in the presence and absence of BPA. Mice were irradiated with neutron beam. The SAS cells were subcutaneously grafted to left hind legs, administered with either BPA or mock treatment, and irradiated with neutron beam.

Neutron irradiation was performed in the Kyoto University Research Reactor (KUR), Japan. Before irradiation, cells were suspended and diluted to 1 × 10^6^ cells/mL. At least 1 h before irradiation, BPA–fructose complex at a boron concentration of 25 ppm [^10^B] or mock control was administrated to the cells. The irradiation doses for BPA-based BNCT were selected according to previous reports [23] and were simulated to be 0, 4, and 24 Gy-Eq for BPA-administered cells. γ-Ray irradiation with ^137^Cs source was carried out using a GammaCell 40 Exactor (Best Theratronics, Kanata, ON, Canada) at the National Cancer Center Research Institute or Nagasaki University (PS-3100SE, Pony Industry, Osaka, Japan), with a dose rate of approximately 100 cGy/min. Cell suspensions in tubes were then irradiated. A schematic diagram of the experiments is shown in Figure 1.

### 2.3. Clonogenic Assay

Following BNCT, the cells were seeded into 6-well plates or 60 mm dishes and cultured for 8–14 days. Colonies were fixed with either 100% (*v*/*v*) methanol or 4% (*v*/*v*) formalin solution and stained with 0.1% crystal violet solution. The number of colonies was counted, and the plating efficiency and surviving fraction were calculated as previously described [9].

### 2.4. ELISA

The cells were seeded and harvested at 6 and 24 h after irradiation. The supernatants and cells were collected separately using ISOGEN (Nippon Gene, Tokyo, Japan). Human/mouse HMGB1 levels were quantified using the HMGB1 ELISA kit II (Shino-Test Corporation, Tokyo, Japan), and mouse HMGB1/HMG-1 ELISA kit (Novus Biologicals, Centennial, CO, USA) according to the manufacturers’ instructions. Recombinant HMGB1 ELISA kit was used as a positive control. The absorbance was measured using a Tecan Safire (Tecan Group AG, Männedorf, Switzerland).

### 2.5. Immunohistochemistry (IHC)

Paraffin-embedded tissues were sectioned and used for the immunofluorescence analysis as previously described [25,26]. Antigen retrieval was performed using low-pH EnVision FLEX Target Retrieval Solution (K8005, Dako, Omnis, Agilent, Santa Clara, CA, USA) according to the manufacturer’s protocol. The sections were blocked with PBS with 10% goat serum (G9023, Sigma-Aldrich, St. Louis, MO, USA) and 10% BSA (010-15153, Wako Pure Chemical Industries, Osaka, Japan) for 2 h at room temperature. After washing twice with PBS, primary antibodies were added (Table S1). The sections were then incubated at 4 °C overnight. These sections were washed six times with PBS and then incubated with the secondary antibody and 4’,6-diamidino-2-phenylindole (for staining of nuclei, 049-18801, Wako Pure Chemical Industries, Osaka, Japan) for 3 h in the dark at room temperature. The samples were then washed six times with PBS and mounted using a mounting medium (VECTASHIELD, H-1000, Vector Laboratories, Burlingame, CA, USA). The antibodies used and the reaction conditions are listed in Table S1. Immunohistochemical observations were performed using an Olympus DP73 fluorescence microscope with 40× objective lens. Images were captured using CellSens imaging software (Olympus, Tokyo, Japan).

### 2.6. Tumorigenesis Analysis and Irradiation

All animal experiments were approved by the Institutional Animal Experiment Committee of Kyoto University and conducted according to the relevant national and international guidelines for animal welfare. All animal studies were performed in accordance with the guidelines of Kyoto University and “Animal Experiments at the National Cancer Center” (approval number T17-052), which meet the ethical standard required by the law and guidelines of experimental animals in Japan. SAS cells were trypsinized and 2 × 10^6^ of them were subcutaneously injected into the left legs of male BALB/c-nu/nu mice (CLEA Japan Inc., Tokyo, Japan). Tumor volumes were calculated using the following formula: (smallest diameter) × (largest diameter) × (height)/2. When the tumor volume reached approximately 100 mm^3^, the mice were administered with BPA–fructose intraperitoneally at 500 mg/kg bodyweight. After 30 min, the mice were then irradiated with BNCT using a thermal neutron beam. BPA–fructose administration without neutron irradiation was used as a mock treatment. The mice were placed in plastic irradiation chambers and then irradiated for 60 min in a heavy water facility at KUR (thermal output of reactor: 1 MW). Li-6-enriched LiF tiles (5 mm thick) were used to shield parts of the body other than the legs. Three days and eight days after irradiation, tumor volumes were measured with a caliper and tumor sections were prepared. Blood samples were collected from the heart under anesthesia.

### 2.7. Statistical Analysis

Statistical analysis was performed with a Student’s *t*-test using JMP software (SAS Institute Inc., Cary, NC, USA). Data are presented as mean ± S.E.

## 3. Results

### 3.1. Effect of BNCT on the Survival of SAS Cells

**Figure 2:**
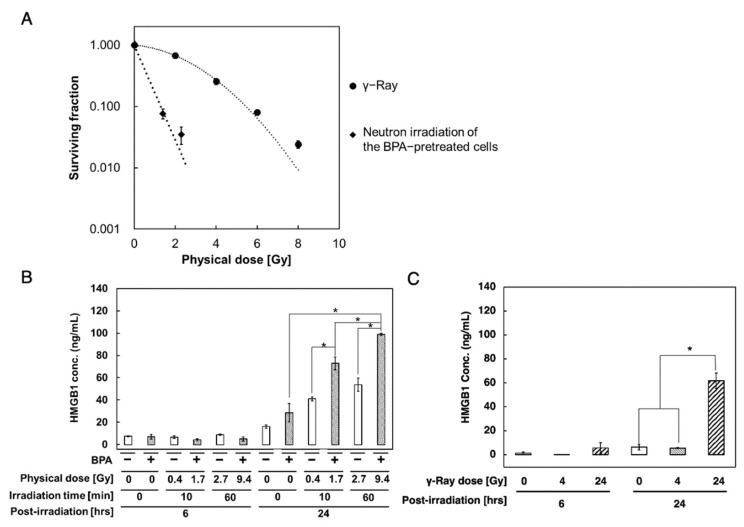
Increased HMGB1 levels in the culture supernatant of SAS cells after neutron beam irradiation with BPA compared with γ-irradiation. (**A**) Cell survival curves of SAS cells after neutron beam irradiation with BPA and γ-irradiation. SAS cells were treated with γ-irradiation (closed circle), neutron irradiation of the BPA-pretreated cells (closed diamond). The surviving fractions were assessed based on colony formation as described in the Materials and Methods. (**B**,**C**) Analysis of the HMGB1 level in the culture supernatant of SAS cells after neutron beam irradiation with BPA (**B**) and γ-irradiation (**C**) with ELISA ((**B**) SHINOTEST, (**C**) Abnova)). BPA at 25 ppm [^10^B] or the control was added 1 h before neutron beam irradiation for BNCT. The background value of the medium was subtracted from each data point. Replicates *n* = 3 in (**A**–**C**). (**B**,**C**) Mean ± S.E. *, *p* < 0.05. Student’s *t*-test.

Previously, we reported that the level of high mobility group box 1 (HMGB1) was elevated in the xenograft of lymphosarcoma cells in vivo 6 h after neutron irradiation in the presence of BPA in the rat model [8]. As HMGB1 has various functions, including transcription factors, ligands for TLR2 and 4, and DAMPs protein, we further analyzed changes in HMGB1 levels in response to BNCT to evaluate the potential of HMGB1 as a biomarker for BNCT responses. The early responses of tumor cells to neutron irradiation, with or without BPA, were investigated in human oral squamous carcinoma SAS cells and xenograft models in the KUR nuclear reactor (Figure 1). SAS cells were irradiated with neutrons, with or without BPA preincubation, or gamma-rays, and cell viability was evaluated using a clonogenic assay (Figure 2A). Neutron beam irradiation after 2 h of BPA preincubation at 25 ppm [^10^B] resulted in lower cell viability in SAS cells compared to that in the absence of BPA and with γ-irradiation. The relative biological effectiveness (RBE) of neutron irradiation with BPA compared to γ-irradiation was approximately 4 at a 10% survival dose.

### 3.2. Enhanced Extracellular HMGB1 Release from BPA-Pretreated SAS Cells after Neutron Irradiation

We set target doses of 24 Gy-Eq (60 min neutron beam irradiation), which is close to the therapeutic doses of BNCT, and 4 Gy-Eq (10 min irradiation) as a lower dose to evaluate the dose-dependent effect of BNCT in BPA-pretreated cells. As shown in Figure 2B, the extracellular HMGB1 level increased in a dose-dependent manner in BPA-pretreated cells 24 h post-neutron irradiation. After 60 min of irradiation, the HMGB1 levels increased by 14-fold at 24 h compared to that at 6 h. An increase in the HMGB1 levels was also observed 24 h after neutron irradiation without BPA; however, it was lower than that observed with BPA. To compare the differences in the cellular responses to radiation responses, we also measured the extracellular release of HMGB1 after γ-irradiation in SAS cells. As shown in Figure 2C, after 4 and 24 Gy γ-irradiation, which are equivalent set doses to the above neutron irradiation conditions, the level of increase was more than 2-fold lower at 24 h with γ-irradiation than that with BNCT.

HMGB1 is released during necrosis in various cell types of cells [27]. In SAS cells, we previously detected increased levels of apoptotic markers, such as the poly(ADP-ribose) polymerase 1 (PARP1) cleavage, 24 h after 60 min of neutron beam irradiation with BPA, accompanying the low-level detection of necrosis associated PARP1 cleavage [9]. Thus, HMGB1 extracellular release may be due to a progression from apoptosis to secondary necrosis in severely damaged cells.

### 3.3. Extracellular HMGB1 Release from BPA-Pretreated A375 Cells after Neutron Irradiation

**Figure 3:**
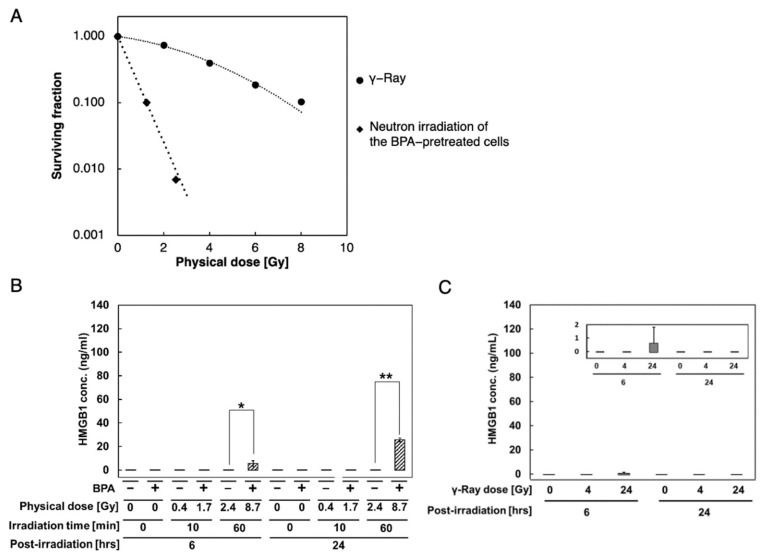
Increased extracellular HMGB1 levels in the culture supernatant of A375 cells after neutron irradiation with BPA. Cell survival curves of A375 cells. (**A**) Cell survival curves of A375 cells. A375 cells treated with γ-irradiation (closed circle) and BPA-pretreated cells irradiated with neutrons (closed diamond) are shown. The surviving fractions were assessed based on colony formation assay. (**B**,**C**) Analysis of HMGB1 levels in the culture supernatant of A375 cells after neutron irradiation with BPA at 25 ppm [^10^B] (**B**) and γ-irradiation (**C**) using ELISA ((**B**) SHINOTEST, (**C**) Abnova). HMGB1 concentration ranged from 0–2 ng/mL. The background value of the medium was subtracted from each data point. The experiments were performed in triplicate. Data are presented as mean ± S.E. *, *p* < 0.05 and **, *p* < 0.005.

We also examined the cellular response to BNCT in human melanoma A375 cells and analyzed HMGB1 levels in the culture supernatant at 6 and 24 h post-neutron irradiation in BPA-pretreated cells. We set target doses of 24 and 4 Gy-Eq, which are equivalent to 60 and 10 min of neutron beam irradiation, respectively. HMGB1 levels in the supernatant of A375 cells were measured using ELISA. As shown in Figure 3B, HMGB1 increased at 6 h after 60 min of neutron irradiation in cells and reached a 5-fold increase at 24 h post-irradiation. In contrast, in the absence of BPA, HMGB1 was not detected 24 h after neutron irradiation. Additionally, we evaluated the extracellular release of HMGB1 after γ-irradiation in A375 cells. HMGB1 levels did not increase in the culture supernatant 24 h after irradiation at 4 and 24 Gy; we detected a low level of HMGB1 at 6 h after 24 Gy irradiation (Figure 3C). Therefore, HMGB1 extracellular release was only observed after neutron irradiation of the BPA-pretreated A375 cells, and the RBE for HMGB1 release is greater than 4, indicating a difference in the cellular responses to neutron irradiation and γ-irradiation in A375 cells.

### 3.4. HMGB1 Distribution in Tumors after BPA Pretreatment and Neutron Irradiation

To investigate the changes in HMGB1 levels in a xenograft model, SAS cells were subcutaneously implanted to the left hind legs of nude mice. Seven days later, mice were administered with BPA–fructose (500 mg/kg body weight, BNCT group) or fructose control, and 30 min later, the left hind legs were irradiated for 60 min with a neutron beam.

**Figure 4:**
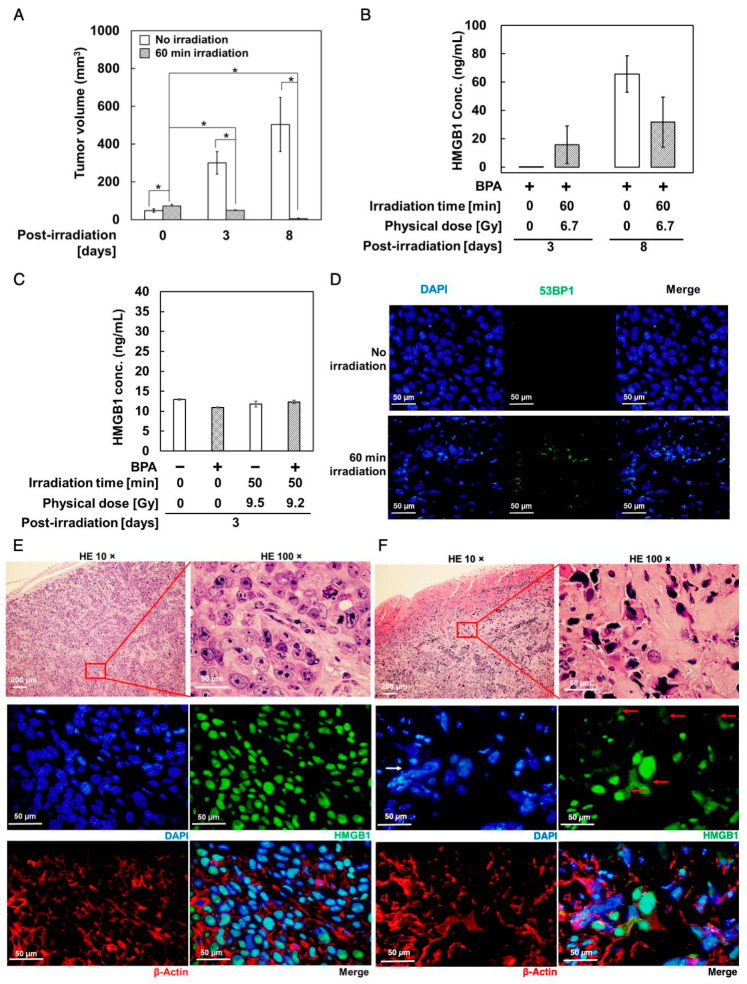
Changes in HMGB1 levels and localization in response to neutron irradiation with BPA in SAS cell-derived tumors. SAS cells were subcutaneously grafted in nude mice and 30 min after administration of BPA–fructose at 500 mg/kg bodyweight, tumors were mock-irradiated (**E**) or locally irradiated for 60 min with neutron beam, ((**F**), BNCT). (**A**) Changes in tumor volumes in response to neutron irradiation after BPA administration. Data are presented as mean ± S.E. *, *p* < 0.05. (**B**) Measurement of plasma levels of human HMGB1 in mice using ELISA (Abnova). (**C**) Mouse plasma HMGB1 levels of C57BL/6 mice without tumor xenograft 3 days after mock irradiation or BNCT measured using ELISA kit (Novus Biologicals). The BNCT group was administered with BPA–fructose at 500 mg/kg bodyweight 30 min before whole-body neutron irradiation. (**D**) Immunostaining of 53BP1 in sections from tumor xenografts of (**A**) at day 3. Bars in (**D**), 50 µm. (**E**,**F**) Immunostaining of the HMGB1 (green) and β-actin (red) in sections from tumor xenograft-bearing mice at day 3. Bars, 200 µm (top, left panel) and 50 µm (other panels). In (**F**), HMGB1 panel, solid red arrow shows the distribution of HMGB1 in the cytoplasm; solid white arrow shows the irregular nuclear morphology. Day 3: mock irradiation, *n* = 7; BNCT group, *n* = 7. Day 8: mock irradiation, *n* = 4; BNCT group, *n* = 4. Physical dose at the skin was estimated to be 6.5 Gy in the BNCT group. For counterstaining of nuclei, 4′,6-diamidino-2-phenylindole (blue) was used.

Compared to non-irradiated controls, the volume of the tumors of neutron-irradiated mice (BNCT group) was significantly decreased on day 3 and continued to decrease, as observed on day 8 (*p* < 0.05, Figure 4A).

Additionally, compared to the control group, the BNCT group showed a higher human plasma HMGB1 level, measured using ELISA, on day 3 (Figure 4B), when tumor growth was significantly retarded compared to the non-irradiated control. Notably, blood HMGB1 levels in the BNCT group on day 8 remained two-fold higher than those on day 3, indicating that the BNCT-induced increase in blood HMGB1 levels can be detected when tumor regression continues. This could be due to the rather stable presence of plasma HMGB1 during shrinkage of the tumor. In the non-irradiated control, there was an increase in HMGB1 levels on day 8 compared to day 3, accompanied by tumor growth, which is consistent with our observation of the extracellular HMGB1 release from untreated SAS cells (Figure 2B). Moreover, we also measured the HMGB1 basal levels in mice without tumor xenografts, using a different ELISA kit, 3 days after whole-body neutron irradiation following BPA administration at 500 mg/kg bodyweight. We did not detect significant changes in plasma HMGB1 levels in the non-irradiated and neutron-irradiated groups (Figure 4C). Therefore, the increase in plasma level in the tumor-bearing BNCT group is due to the extracellular HMGB1 release during cell death.

To examine the HMGB1 distribution in tumor tissues, tumor sections were prepared and analyzed using immunostaining 3 days after irradiation. Reportedly, 53BP1 localizes at DSBs and forms foci after particle beam irradiations including BNCT [4]. To confirm DSB damage, we analyzed 53BP1 foci formation in the tumor sections on day 3. As shown in Figure 4D, 53BP1 foci were observed in the tumors irradiated with BPA pretreatment and a neutron beam, but not in the non-irradiated control, indicating that DSB damage was induced only in the irradiated group. Sections from non-irradiated controls showed exclusive localization of HMGB1 in the nuclei of undamaged cells (Figure 4E). In contrast, the tumor sections from the BNCT group showed localization of HMGB1 in both the nuclei and the surrounding cytoplasmic regions in the cells, especially those with a larger size and irregular nuclear morphology (Figure 4F), where pyknotic cells were abundantly observed, suggesting HMGB1 release in the cytoplasm from dying/dead cells.

## 4. Discussion

In this study, we demonstrated that neutron irradiation of BPA-pretreated human melanoma A375 and oral squamous carcinoma SAS cells at a therapeutic dose induces an early induction of a high level of extracellular HMGB1 release, compared with the equivalent dose of γ-irradiation. In the xenograft model of SAS cells in nude mice, HMGB1 was detected in plasma 3 days post-BNCT irradiation, even when the tumor regression started.

Furthermore, HMGB1 levels remained high 8 days post-BNCT irradiation, indicating that plasma HMGB1 can still be detected even if the tumors decrease in size. Therefore, plasma HMGB1 levels are a potential early biomarker for response to BNCT.

However, our observation was different from a previous report that showed the shorter half-life of HMGB1 in human serum [28]. The half-life of HMGB1 is significantly affected by the extracellular environment such as hypoxic environment and high tumor cell densities [29]. Therefore, our experimental conditions may have extended the half-life of HMGB1.

Additionally, in neutron-irradiated BPA-pretreated SAS and A375 cells, we observed that cell death and extracellular HMGB1 release increased 24 h post-irradiation. Tumor regression was observed from days 3–8 (Figure 4A). Therefore, in this study we measured cell damage and extracellular HMGB1 levels on day 3 and 8 post-irradiation (Figure 4B). Our immunostaining showed that HMGB1 localization changed from the nuclei to the surrounding cytoplasmic regions after irradiation with neutrons, which is possibly indicative of cell death. We also noted that the HMGB1 basal level in mice did not change 3 days after whole-body neutron irradiation after administration of BPA, suggesting that HMGB1 release from normal tissue cells was not induced after BNCT. Thus, HMGB1 plasma level could possibly be used as a biomarker for monitoring the early tumor response to BNCT.

Dying tumor cells are reported to promote repopulation of the tumors by activating caspase-3 after photon beam irradiation [30]. Apoptosis and necrosis have been reported to be involved in tumor repopulation [31]. We previously reported that apoptosis with caspase-3 activation and PARP1 cleavage was mainly observed after neutron irradiation in BPA-treated SAS cells. However, necrotic PARP1 cleavage was also observed within 24 h after 24 Gy-Eq neutron irradiation of these cells [9], where the extracellular release of HMGB1 could possibly be induced by the secondary necrosis by shifting from apoptosis in the severely damaged cells. BNCT may also directly induce both apoptosis and necrosis [32]. However, whether HMGB1 release is involved in tumor repopulation at the early and late responses to BNCT should be evaluated in future studies in xenograft models.

Necrotic cells and released DAMPs are immunogenic and evoke diverse inflammatory reactions [33]. He et al. showed that HMGB1 released by irradiated tumor cells promotes the proliferation of cancer cells, which was abolished by HMGB1 inhibitors and knocked down of HMGB1. Therefore, the extracellular HMGB1 release from tumor cells exposed to photon beam irradiation or chemotherapy has been proposed as a therapeutic and prognostic biomarker [31]. However, it is also reported that HMGB1 binds to other DAMPs molecules and stimulates diverse signaling pathways [34]. In our study, SAS cells showed a much higher level of HMGB1 release than A375 cells, suggesting that HMGB1 levels might differ among individual tumors. Therefore, the HMGB1 release pattern and its effects should be studied in various types of cancers using in vitro and in vivo models.

Current evidence suggests that extracellular HMGB1 release may act systemically as well as locally in the tumor microenvironment to promote tumor cell survival and inflammatory reactions in a paracrine manner. Ethyl pyruvate and glycyrrhizin inhibit the action of HMGB1 and mitigate acetaminophen-induced hepatotoxicity [35]. However, these inhibitors reduce the repopulation of tumor cells after X-ray irradiation [31]. Although ethylpyruvate and glycyrrhizin are not specific inhibitors for HMGB1, it is an open question whether these inhibitors effectively decrease the tumor cell proliferation after BNCT irradiation by inhibiting HMGB1 function.

## 5. Conclusions

In this study, we analyzed the release of HMGB1 from cultured tumor cells and an in vivo model of xenografted human squamous cell tumors in nude mice, in response to BPA pretreatment and neutron irradiation. We showed that plasma HMGB1 increased in the early response stage and was stably detected during tumor regression for at least 8 days. On the other hand, HMGB1 is normally detected at low levels in the plasma of untreated patients with cancer [36] or is overexpressed in some tumors [37]; therefore, the changes in HMGB1 levels in plasma of human patients should also be investigated to further validate the significance of changes in HMGB1 levels as a therapeutic biomarker for BNCT.

## Data Availability

Not applicable.

## Supplementary Materials

The following are available online at https://www.mdpi.com/2079-7737/11/3/420/s1, Figure S1: Calibration curves for HMGB1 ELISA experiments. Representative calibration curves for HMGB1 ELISA kits (Shino-Test (for human and mouse HMGB1), Abnova (for human HMGB1), and Novus Biologicals (for mouse HMGB1)) are shown. Absorbances measured at 450 nm after subtraction of those measured at 620 nm are shown. Table S1: Antibodies used for immunohistochemistry.
