# Correction: Imamichi et al. Extracellular Release of HMGB1 as an Early Potential Biomarker for the Therapeutic Response in a Xenograft Model of Boron Neutron Capture Therapy. *Biology* 2022, *11*, 420

**DOI:** 10.3390/biology12081112

**Published:** 2023-08-10

**Authors:** Shoji Imamichi, Lichao Chen, Tasuku Ito, Ying Tong, Takae Onodera, Yuka Sasaki, Satoshi Nakamura, PierLuigi Mauri, Yu Sanada, Hiroshi Igaki, Yasufumi Murakami, Minoru Suzuki, Jun Itami, Shinichiro Masunaga, Mitsuko Masutani

**Affiliations:** 1 Department of Molecular and Genomic Biomedicine, School of Biomedical Sciences, Nagasaki University Graduate, Nagasaki 852-8523, Japan; simamich@ncc.go.jp (S.I.); chen202107@outlook.com (L.C.); y-tong@nagasaki-u.ac.jp (Y.T.); takae-o@nagasaki-u.ac.jp (T.O.); jj20210059@ms.nagasaki-u.ac.jp (Y.S.); 2 Lab of Collaborative Research, Division of Cellular Signaling, National Cancer Center Research Institute, Tokyo 104-0045, Japan; j8313605@ed.tus.ac.jp; 3 Central Radioisotope Division, National Cancer Center Research Institute, Tokyo 104-0045, Japan; 4 Division of BNCT, EPOC, National Cancer Center, Tokyo 104-0045, Japan; satonaka@ncc.go.jp (S.N.); hirigaki@ncc.go.jp (H.I.); jitami@ncc.go.jp (J.I.); 5 Department of Biological Science and Technology, Faculty of Industrial Science and Technology, Tokyo University of Science, Tokyo 125-8585, Japan; yasufumi@rs.noda.tus.ac.jp; 6 Department of Radiation Oncology, National Cancer Center Hospital, Tokyo 104-0045, Japan; 7 Clinical Proteomics Laboratory, Institute of Biomedical Technologies, National Research Council, 93-20054 Milan, Italy; pierluigi.mauri@itb.cnr.it; 8 Institute for Integrated Radiation and Nuclear Science, Kyoto University, Kumatori 590-0494, Japan; sanada.yu.6n@kyoto-u.ac.jp (Y.S.); suzuki.minoru.3x@kyoto-u.ac.jp (M.S.); masunaga.shinichiro.6m@kyoto-u.jp (S.M.)

## 1. Corrected in Front Matter

In the original publication [1], The previous mailing address of the fourth author is no longer used, the correct email address is y-tong@nagasaki-u.ac.jp. Tasuku Ito and Yuka Sasaki contributed equally to this work, which should have been removed during proofreading stage. The symbol “‡” after their names should have been removed accordingly. The corrected Front Matter appears above.

## 2. Error in Figure

In the original publication [1], there was a mistake in Figure 4F as published. In Figure 4F, the left bottom photo of β-actin staining was replaced by the photo of β-actin staining of the left bottom photo of Figure 4E during the proof preparation process. The corrected Figure 4F appears below.

The authors state that the scientific conclusions are unaffected. This correction was approved by the Academic Editor. The original publication has also been updated.

## Figures and Tables

**Figure 4:**
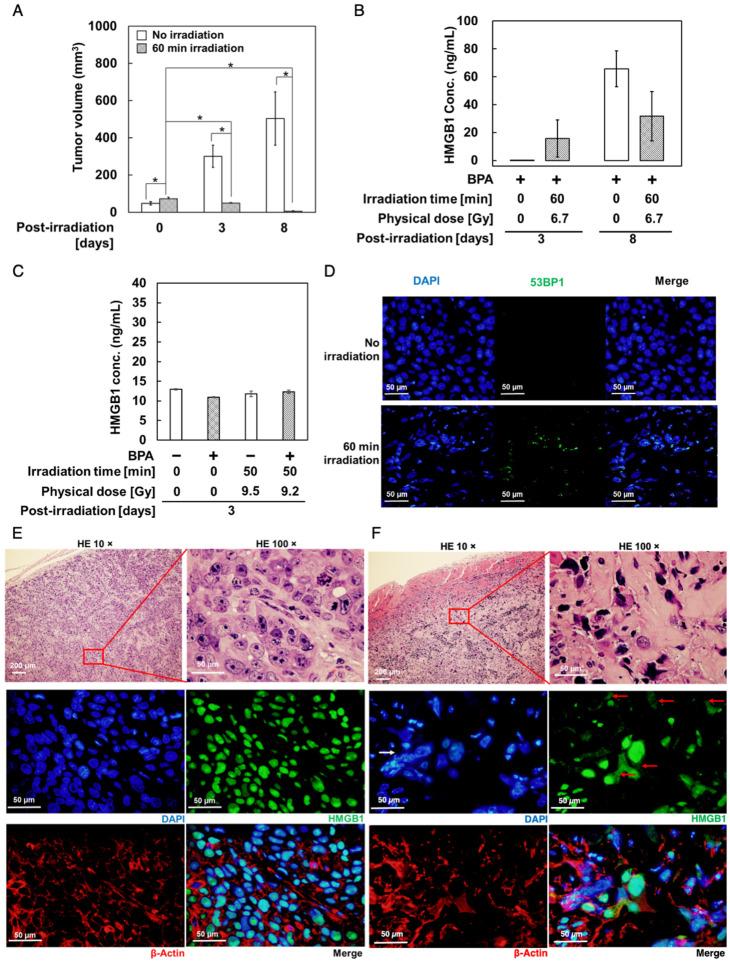
Changes in HMGB1 levels and localization in response to neutron irradiation with BPA in SAS cell-derived tumors. SAS cells were subcutaneously grafted in nude mice and 30 min after administration of BPA–fructose at 500 mg/kg bodyweight, tumors were mock-irradiated (**E**) or locally irradiated for 60 min with neutron beam, ((**F**), BNCT). (**A**) Changes in tumor volumes in response to neutron irradiation after BPA administration. Data are presented as mean ± S.E. *, *p* < 0.05. (**B**) Measurement of plasma levels of human HMGB1 in mice using ELISA (Abnova). (**C**) Mouse plasma HMGB1 levels of C57BL/6 mice without tumor xenograft 3 days after mock irradiation or BNCT measured using ELISA kit (Novus Biologicals). The BNCT group was administered with BPA–fructose at 500 mg/kg bodyweight 30 min before whole-body neutron irradiation. (**D**) Immunostaining of 53BP1 in sections from tumor xenografts of (**A**) at day 3. Bars in (**D**), 50 µm. (**E**,**F**) Immunostaining of the HMGB1 (green) and β-actin (red) in sections from tumor xenograft-bearing mice at day 3. Bars, 200 µm (top, left panel) and 50 µm (other panels). In (**F**), HMGB1 panel, solid red arrow shows the distribution of HMGB1 in the cytoplasm; solid white arrow shows the irregular nuclear morphology. Day 3: mock irradiation, *n* = 7; BNCT group, *n* = 7. Day 8: mock irradiation, *n* = 4; BNCT group, *n* = 4. Physical dose at the skin was estimated to be 6.5 Gy in the BNCT group. For counterstaining of nuclei, 4′,6-diamidino-2-phenylindole (blue) was used.
