# Supplementary material for: Extracellular Release of HMGB1 as an Early Potential Biomarker for the Therapeutic Response in a Xenograft Model of Boron Neutron Capture Therapy
Source: Biology (Basel). 2022 Mar 10;11(3):420. doi: 10.3390/biology11030420 (PMC8945761; doi:10.3390/biology11030420)
Supplement: Supplementary file 1 [file biology-11-00420-s001.zip › biology-1554804-supplementary.pdf]

Supplementary file

Table S1. Antibodies used for immunohistochemistry.

Antibody	Host	Species reactivity	Company	Cat#
HMGB1	Rabbit/polyclonal	Human/mouse	Abcam	ab18256
Anti-53BP1	Rabbit/polyclonal	Human	Bethyl	A300-272A
β-Actin	Mouse/monoclonal	Human/mouse	Sigma-Aldrich	A2228
Anti-Rabbit IgG, AF488, Secondary Antibody	Goat/polyclonal	Rabbit	Invitrogen	A11008
Anti-mouse IgG, AF594, Secondary Antibody	Goat/polyclonal	Mouse	Invitrogen	A32742

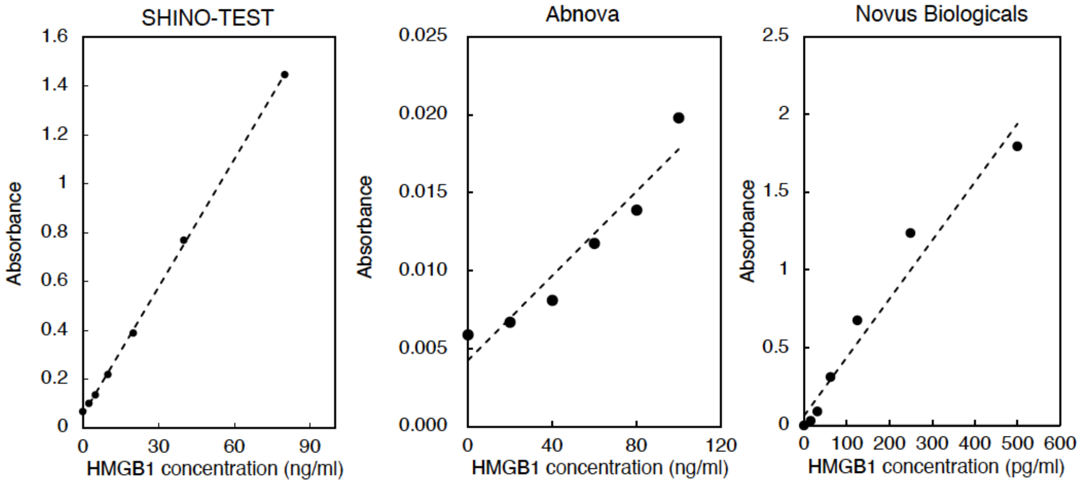

Figure S1. Calibration curves for HMGB1 ELISA experiments. Representative calibration curves for HMGB1 ELISA kits (SHINO-TEST (for human and mouse HMGB1), Abnova (for human HMGB1), and Novus Biologicals (for mouse HMGB1)) were shown. Absorbances were measured at 450 nm after subtraction of those measured at 620 nm are shown.
